# Potential vulnerability and resilience to accelerated brain aging in women exposed to stressful life events: insights from the brain age prediction model

**DOI:** 10.1016/j.ynstr.2025.100763

**Published:** 2025-09-23

**Authors:** Hyeonseok Jeong, Yoonji Joo, Youngeun Shim, Yejin Kim, Hyeonji Lee, Yunjung Jin, Seog Ju Kim, Sujung Yoon, In Kyoon Lyoo

**Affiliations:** a Ewha Brain Institute, Ewha Womans University, Seoul, South Korea; b Department of Brain and Cognitive Sciences, Ewha Womans University, Seoul, South Korea; c Department of Psychiatry, Samsung Medical Center, Sungkyunkwan University College of Medicine, Seoul, South Korea; d Graduate School of Pharmaceutical Sciences, Ewha Womans University, Seoul, South Korea

**Keywords:** Brain age, Stress, Emotional symptom, Alcohol-use symptom, Resilience

## Abstract

Brain age prediction models consistently reveal accelerated brain aging in psychiatric disorders, yet associations with stress, independent of formal psychiatric diagnoses, remain uncertain. This study investigated the relationships of emotional and alcohol-use symptoms, common and often comorbid stress-related symptoms, and resilience with brain aging using high-resolution structural MRI data from 520 women who experienced stressful life events. Participants were divided into four groups based on the presence of emotional and alcohol-use symptoms: no symptoms (n = 287), emotional symptoms only (n = 93), alcohol-use symptoms only (n = 79), or both symptoms (n = 61). Individual brain age gap (BAG)—the difference between predicted brain age and chronological age—was calculated using a deep learning-based brain age prediction model. Individual and interactive associations of the presence of two symptoms with BAG were assessed using two-way ANCOVA. Relationships of a continuous composite symptom score integrating both symptoms and resilience with BAG were evaluated. Participants with both symptoms exhibited significantly larger BAG than the other groups, with a statistically significant interaction between two symptom domains (p = 0.017). Across the full sample, composite symptom scores were positively associated with BAG (β = 0.16, p = 0.004), with an even stronger association within individuals with both symptoms (β = 0.34, p < 0.001). Conversely, higher resilience was linked to smaller BAG across all participants (β = −0.10, p = 0.046). The negative association between resilience and BAG was statistically mediated by the composite symptom score (b = −0.011, p = 0.010). These findings may suggest a synergistic, more-than-additive association between stress-related symptoms and accelerated brain aging, as well as a potentially buffering association of resilience.

## Highlights

• Associations of BAG with stress symptoms and resilience were assessed in women.
• A deep learning model estimated BAG from structural MRI data.
• Co-occurring emotional and alcohol-use symptoms were linked to larger BAG.
• Composite symptom severity showed a positive association with BAG.
• Resilience was negatively correlated with BAG with statistical mediation by symptoms.

## 1 Introduction

Stressful life events (SLE), encompassing interpersonal, financial, occupational, and traumatic stressors, are well-established contributors to mental health challenges, often precipitating the development of psychiatric disorders such as depression, anxiety, and substance use disorders (Diez-Canseco et al., 2024; Keyes et al., 2011; Tibubos et al., 2021). Additionally, these stressors have been linked to structural and functional abnormalities in the brain (McEwen et al., 2016; McManus et al., 2022). Exposure to stress is also associated with accelerated biological aging, including telomere shortening and epigenetic aging (Simons et al., 2016; Zannas et al., 2015). Chronic stress is implicated in neurodegenerative changes and an elevated risk of mild cognitive impairment and dementia through mechanisms such as neuronal loss, β-amyloid plaque accumulation, tau protein phosphorylation, and neuroinflammation, indicating its impact on brain aging (Franks et al., 2021; Martisova et al., 2013). Conversely, resilience, defined as the dynamic process of adaptive overcoming and recovery in the face of stress and adversity (Wu et al., 2013), is associated with a reduced risk of maladaptive brain responses and mental disorders following adverse life events, highlighting its potential as a protective factor (Roeckner et al., 2021; Sheerin et al., 2018).

Recent advancements in machine learning-based brain age prediction models have provided valuable tools to investigate the influence of environmental factors and disease states on brain aging (Cole et al., 2019). Brain age gap (BAG), defined as the difference between an individual's estimated brain age from neuroimaging data and their chronological age, serves as a practical indicator of deviations from normative brain aging patterns (Cole et al., 2019). Its clinical relevance has been explored across diverse populations (Cole et al., 2019). Notably, psychiatric conditions, including emotional disorders and substance use disorders, have been associated with accelerated brain aging, as evidenced by increased BAG (Clausen et al., 2022; Han et al., 2021a; Scheffler et al., 2024; Stratton et al., 2024). While a few studies have identified significant associations between exposure to SLE or long-term depressive symptoms and increased brain age relative to chronological age in adults (Dintica et al., 2023; Hatton et al., 2018; Mareckova et al., 2023), the specific impact of key stress-related symptoms—such as emotional and alcohol-use symptoms (Castillo-Carniglia et al., 2019; Kaufman and Charney, 2000)—and resilience on brain aging remains to be fully elucidated.

Emerging evidence suggests that women may be more vulnerable than men to the effects of stress on the brain and mental health, influenced by neurobiological, hormonal, and sociocultural factors (Edwards et al., 2023; Li and Graham, 2017; McManus et al., 2022). Throughout the lifespan, higher stress levels are more strongly associated with a greater prevalence of psychiatric disorders and more pronounced cognitive decline in women compared to men (Li and Graham, 2017; McManus et al., 2022). Additionally, animal studies have shown that acute stress increases β-amyloid levels in female mice but not in male mice (Edwards et al., 2023), suggesting that stress-related advanced brain aging may be more severe and clinically significant in women.

The present study aimed to investigate the associations of emotional and alcohol-use symptoms, two common and often comorbid stress-related symptoms (Castillo-Carniglia et al., 2019; Kaufman and Charney, 2000), as well as resilience with brain aging in a community-based sample of women with SLE. Given that participants were not recruited based on psychiatric diagnoses, our primary analysis focused on whether the presence of clinically significant emotional and alcohol-use symptoms was independently or jointly associated with BAG. To complement this approach, we conducted a secondary analysis evaluating dose-dependent patterns through linear associations of BAG with a continuous composite score reflecting overall symptom severity, as well as with resilience levels. Lastly, given prior evidence of inverse relationships between resilience and stress-related symptoms as well as brain structural and functional characteristics (Sheerin et al., 2018; Wu et al., 2013), we tested whether emotional and alcohol-use symptom severity statistically account for the association between resilience and BAG.

## 2 Materials and methods

### 2.1 Participants

Adult women, aged between 18 and 64 years, who had experienced at least one SLE in the past year, were recruited from the community for this study. Participants completed a structured self-report checklist to report SLE experienced in the past year. This checklist presented predefined categories of SLE types, each accompanied by illustrative examples to aid clarification and accurate self-identification. Participants were instructed to select all applicable categories they had experienced. The structured checklist included four primary categories with corresponding examples: (a) personal and family-related stressors, including loss of a loved one, divorce or separation, marital conflict, parenting challenges, serious illness or injury of self or family members, caring for a sick or elderly family member, infertility, miscarriage, or major family disputes; (b) work and financial stressors, encompassing job loss, unemployment, workplace harassment or conflict, significant financial hardship, bankruptcy, retirement, major work-related transitions, or career setbacks; (c) social and environmental stressors, including residential relocation, discrimination based on race, gender, or other characteristics, social harassment, social isolation, community violence exposure, or major changes in social support networks; and (d) traumatic events, such as natural disasters, serious accidents, violence or abuse, witnessing violence, being a victim of crime, physical or sexual assault, life-threatening experiences, or exposure to combat or war-related events.

The diagnosis of psychiatric disorders was conducted using the Structured Clinical Interview for DSM-5. Participants were included regardless of the presence of current psychiatric disorders. Exclusion criteria consisted of serious medical or neurological conditions, structural brain abnormalities or lesions detected on MRI, and contraindications to undergoing brain MRI scans. Screening procedures involved comprehensive clinical interviews, medical history reviews, routine laboratory tests, and physical and mental status examinations. The study protocol was approved by the Institutional Review Board of Ewha Womans University, and all participants provided written informed consent prior to participation.

### 2.2 Clinical assessments

Participants were categorized based on the presence of two symptom domains: emotional symptoms and alcohol-use symptoms. Emotional symptoms were considered present if participants exhibited depressive or anxiety symptoms, which were assessed using the Hamilton Depression Rating Scale (HDRS) (Hamilton, 1960) and the Hamilton Anxiety Rating Scale (HARS) (Hamilton, 1959), respectively. A cut-off score of ≥8 was applied for both scales (Matza et al., 2010; National Collaborating Centre for Mental Health, 2010). The presence of alcohol-use symptoms was determined using the Alcohol Use Disorders Identification Test (AUDIT) with a cut-off score of ≥8 (Conigrave et al., 1995; Saunders et al., 1993). Based on these criteria, participants were classified into four mutually exclusive subgroups: (1) Reference group (n = 287) with neither emotional nor alcohol-use symptoms; (2) Group A (n = 93) with emotional symptom only; (3) Group B (n = 79) with alcohol-use symptom only; and (4) Group C (n = 61) with both emotional and alcohol-use symptoms. Levels of resilience were assessed using the Connor-Davidson Resilience Scale (CD-RISC), with higher scores indicating greater resilience (Connor and Davidson, 2003).

### 2.3 Brain image acquisition

Brain MRI scans were acquired using a 3.0 T Philips MR scanner (Philips Healthcare, Best, The Netherlands) with a 32-channel head coil. High-resolution three-dimensional T1-weighted images were obtained using the following parameters: repetition time (TR) = 7.4 ms, echo time (TE) = 3.4 ms, field of view (FOV) = 224 × 224 mm^2^, reconstruction voxel size = 1 × 1 × 1 mm^3^, flip angle = 8°, slice thickness = 1 mm, with 180 sagittal slices. To ensure the quality of the neuroimaging data, a rigorous screening protocol was implemented. All T1-weighted images were visually inspected by experienced radiologists. Scans revealing clinically significant structural abnormalities, poor image quality, or excessive motion artifacts were excluded from all subsequent analyses.

### 2.4 Brain age prediction model

This study employed a previously validated brain age prediction model based on convolutional neural network (CNN) and multi-layer perceptron (MLP) algorithms, originally developed using 3004 open-source datasets (Joo et al., 2023). Details on the original model are provided elsewhere (Joo et al., 2023). Although this model demonstrated sufficient validity, with a mean absolute errors (MAE) of 4.910 (SD = 3.703) years in the external validation, we retrained and optimized it using an independent dataset of healthy Korean individuals to enhance the accuracy of brain age estimation for the current Korean population.

The dataset comprised 4087 T1-weighted images from healthy Korean adults without any radiological abnormalities or significant artifacts (mean age = 39.7 years, SD = 13.4 years, range = 18.6–79.8 years, 46.5 % male), independent of both the cohort used in the original model (Joo et al., 2023) and the participants in the current study. The data were randomly divided into training (n = 3269) and test (n = 818) sets in an 8:2 ratio. Age and sex information for the dataset is provided in Supplementary Table 1. The same preprocessing procedures as those applied in the original model were used (Joo et al., 2023). T1-weighted images were preprocessed using the unified segmentation approach implemented in Statistical Parametric Mapping (SPM) 12 software (Wellcome Centre for Human Neuroimaging, London, UK). Each image was segmented into different tissue types, corrected for magnetic field inhomogeneities, non-linearly registered to the Montreal Neurological Institute (MNI) standard template, and resampled to an isotropic voxel size of 1.5 mm using cubic spline interpolation, resulting in a bias-corrected and MNI-registered T1-weighted image with a field-of-view of 105 × 127 × 105.

The CNN architecture consisted of sequential convolutional blocks, each comprising a 3D convolution layer, batch normalization, a rectified linear unit (ReLU) activation function, and a max pooling layer with a stride of two. The initial block included eight feature channels, with subsequent blocks doubling the number of channels to enhance the model's capacity to capture complex brain structures. The final output of the convolutional blocks was flattened and passed through a dense layer with 64 neurons activated by ReLU. This was followed by a batch normalization layer, a dropout layer with a rate of 0.3, and another dense layer with 16 neurons, also utilizing ReLU activation. The MLP component processed categorical sex information using a dense layer with 16 neurons (ReLU activation), followed by a dense layer with 4 neurons (ReLU activation). The outputs from the CNN and MLP components were merged via a concatenation layer and processed through a dense layer with 4 neurons (ReLU activation). The final brain age prediction was generated through a dense layer with one neuron applying a linear activation function. Model optimization involved hyperparameter tuning, and performance evaluation was conducted using the test dataset. The Adam optimizer, configured with a learning rate of 0.01 and a decay rate of 0.003, yielded the best results. All network architectures were implemented in Python 3.9 using TensorFlow 2.10, which includes Keras 2.10. Additional analyses, including model evaluation, were performed using scikit-learn 1.3. All computations were conducted on a computational environment equipped with two NVIDIA Titan Xp GPUs (12 GB each). The MAE for the test set was 3.507 (SD = 2.931) years. Pearson's correlation coefficient between predicted brain age and chronological age was 0.941 (p < 0.001, R^2^ = 0.886) for the test set.

The BAG was calculated as the difference between the estimated brain age and chronological age, with a positive BAG indicating the accelerated brain aging relative to chronological age. It is well-established that neuroimaging-based brain age prediction models inherently exhibit a systemic, age-dependent bias attributable to the regression to the mean phenomenon. Specifically, brain age tends to be overestimated in younger individuals and underestimated in older individuals, with higher accuracy for those near the mean age of the training dataset (de Lange and Cole, 2020). To address this methodological challenge, we chose to include chronological age as a covariate in all statistical analyses. While alternative methods such as applying an explicit age-bias correction exist, the choice of a specific correction technique can influence both model performance and statistical outcomes (de Lange et al., 2022; More et al., 2023). In line with current best practices in the field (Clausen et al., 2022; de Lange and Cole, 2020; Han et al., 2021a; Scheffler et al., 2024), including age as a covariate is considered an equally effective and transparent approach. This method not only corrects for the age-dependent bias in the BAG but also simultaneously adjusts for the influence of age on other variables in the models, thus providing a more robust statistical framework.

### 2.5 Statistical analysis

The normality of the data distribution was assessed with Shapiro–Wilk test. Comparisons of continuous demographic and clinical variables among the four subgroups were conducted using one-way analysis of variance (ANOVA) followed by post hoc pairwise comparisons adjusted for multiple comparisons using the Benjamini–Hochberg false discovery rate (FDR) method. Categorical variables were analyzed using Chi-square test or Fisher's exact test, as appropriate.

The main and interactive associations of emotional and alcohol-use symptom presence with BAG were assessed using a two-way analysis of covariance (ANCOVA), with age included as a covariate. Post-hoc pairwise comparisons were adjusted using the Benjamini–Hochberg FDR method. The addition of both linear and quadratic age terms did not significantly improve model fit (likelihood ratio test: χ^2^ = 2.25, p = 0.134); thus, only a linear age term was included in the final model.

To evaluate the dose-dependent relationships between overall severity of emotional and alcohol-use symptoms and BAG, we defined composite symptom scores integrating both symptoms. Raw HDRS, HARS, and AUDIT scores were standardized as z-scores using the mean and standard deviation of the Reference group. The emotional symptom score of each individual was calculated as the mean of standardized HDRS and HARS scores, while alcohol-use symptom score was represented by the standardized z-scores of the AUDIT. Multiple linear regression analyses were conducted to examine the association between BAG and composite symptom score or CD-RISC score across the entire sample, as well as within individual subgroups, with adjustment for age.

We conducted a mediation analysis to test the hypothesis that composite symptom severity mediates the relationship between resilience and BAG. In this model, the CD-RISC score served as the independent variable (X), the composite symptom score as the mediator (M), and BAG as the dependent variable (Y), while controlling for chronological age as a covariate. The significance of the indirect effect (the path from X to Y through M) was evaluated.

To evaluate the potential confounding effects of cardiovascular health and socioeconomic status (SES) on BAG (Busby et al., 2023; Hatton et al., 2018; McAvoy et al., 2025), sensitivity analyses were performed by repeating all primary analyses, with each of following incorporated as a covariate individually: the presence of specific cardiovascular risk factors, including hypertension, hyperlipidemia, diabetes, obesity (body mass index ≥25) (Haam et al., 2023), and smoking (Hajar, 2016); total count of cardiovascular risk factors; or SES, assessed with Four-Factor Hollingshead SES Scale (Hollingshead, 2011). We further re-estimated the models including each SLE type to account for significant between-group differences in SLE categories: because some participants experienced more than one SLE category, each SLE type was entered into a separate model as a binary covariate (experienced vs not). Additionally, we repeated the main analyses to assess the robustness of our findings by excluding Reference-group participants with psychiatric diagnoses or potential outliers with BAG values > 20 or < −20 years.

To examine whether BAG varied according to the severity and accumulation of SLE, we conducted two sets of analyses: (1) comparisons of BAG between participants who experienced each specific SLE type versus those who did not, and (2) comparisons of BAG between participants who experienced a single SLE type versus those who experienced multiple SLE types. All analyses were performed using multiple linear regression analyses, with adjustments for age.

For all regression and mediation analyses, statistical inference was performed using bootstrapping with 5000 resamples to obtain robust estimates and confidence intervals (Delucchi and Bostrom, 2004). A two-tailed p value < 0.05 was considered statistically significant. All statistical analyses were performed using STATA version 19 (StataCorp., College Station, TX, USA).

## 3 Results

### 3.1 Demographic and clinical characteristics

A total of 520 women with SLE were included in the analysis. Demographic characteristics of the study participants are presented in Table 1. The mean age of the sample was 38.6 years (SD = 9.1 years; range, 18.6–64.4 years). Significant differences in chronological age were observed among the four groups (F (3,516) = 5.23, p = 0.002). Post-hoc analyses, adjusted for multiple comparisons, revealed that participants in the Group C were significantly younger than those in the Group A and the Reference group. Similarly, the Group B was younger than the Group A. No significant age differences were observed among other group comparisons. Years of education differed significantly among the four groups (F (3,516) = 13,99, p < 0.001), with post-hoc tests indicating that the Reference group had higher education levels than all other groups, and the Group B had higher levels than the Group A. There was a significant group difference in SES (χ^2^ (6) = 49.54, p < 0.001). Post-hoc pairwise comparisons revealed that the SES distribution of the Reference group was significantly different from that of Group A and Group C.

**Table 1:** Demographic characteristics of study participants.

Characteristics	Reference group	Group A	Group B	Group C	Total	Test^a^
N	287	93	79	61	520	
Age	39.0 (7.5)	40.8 (11.6)	37.7 (8.5)	35.2 (11.3)	38.6 (9.1)	F (3,516) = 5.23, p = 0.002
Education (year)	15.6 (2.1)	13.8 (3.2)	14.9 (2.7)	14.3 (1.9)	15.0 (2.5)	F (3,516) = 13.99, p < 0.001
Socioeconomic status						χ^2^ (6) = 49.54, p < 0.001
Upper class	223 (77.7 %)	44 (47.3 %)	51 (64.6 %)	29 (47.5 %)	347 (66.7 %)	
Middle class	51 (17.8 %)	29 (31.2 %)	21 (26.6 %)	20 (32.8 %)	121 (23.3 %)	
Lower class	13 (4.5 %)	20 (21.5 %)	7 (8.9 %)	12 (19.7 %)	52 (10.0 %)	
Type of stressful life event^b^						
Personal and family-related stressors	243 (84.7 %)	51 (54.8 %)	58 (73.4 %)	29 (47.5 %)	381 (73.3 %)	χ^2^ (3) = 55.79, p < 0.001
Work and financial stressors	26 (9.1 %)	20 (21.5 %)	10 (12.7 %)	12 (19.7 %)	68 (13.1 %)	χ^2^ (3) = 12.23, p = 0.007
Social and environmental stressors	5 (1.7 %)	11 (11.8 %)	2 (2.5 %)	11 (18.0 %)	29 (5.6 %)	χ^2^ (3) = 34.28, p < 0.001
Traumatic events	68 (23.7 %)	62 (66.7 %)	25 (31.7 %)	47 (77.1 %)	202 (38.9 %)	χ^2^ (3) = 97.24, p < 0.001

Data are presented as n (%) or mean (standard deviation). Groups were defined as: Reference group (no symptoms), Group A (emotional symptoms only), Group B (alcohol-use symptoms only), and Group C (both symptoms).

a One-way ANOVA or Chi-square tests were conducted.

b Participants who fell into more than one category were counted multiple times. P-values for the four stressful life event types were adjusted using the Benjamini–Hochberg false discovery rate correction.

Among the four types of SLE, personal and family-related stressors were the most common (73.1 %), followed by traumatic events (38.9 %), work and financial stressors (13.1 %), and social and environmental stressors (5.6 %) across all participants. Significant differences in the types of SLE were observed among the four subgroups (all p < 0.05). Specifically, personal and family-related stressors were most prevalent in the Reference group, followed by the Group B. Although both the Group A and Group C exhibited lower frequencies of these stressors, the differences between these two groups were not statistically significant. In contrast, work and financial stressors were more frequent in the Group A and Group C compared to the Reference group. No significant differences were observed among other groups. Regarding social and environmental stressors as well as traumatic events, both the Group A and Group C demonstrated higher frequencies compared to the Reference group and Group B. However, differences between the Reference group and Group B, as well as between the Group A and Group C, were not statistically significant.

The prevalence of psychiatric disorders among participants was presented in Supplementary Table 2. Significant group differences were observed in the prevalence of anxiety, depressive, and alcohol use disorders among the four subgroups (all p < 0.05). Specifically, higher frequencies of anxiety and depressive disorders were found in the Group A and Group C compared to the Reference group and Group B. However, differences between the Reference group and Group B, and between the Group A and Group C, were not significant. Regarding alcohol use disorder, the Group C demonstrated a higher prevalence compared to the Reference group and Group A. Additionally, the Group B had more participants with alcohol use disorder than the Reference group. No other pairwise comparisons revealed statistically significant differences. Although 22 participants in the Reference group had mental disorders, their depressive, anxiety, and alcohol-use symptoms were below the cutoff scores on the HDRS, HARS, and AUDIT, respectively. Individual scale scores, standardized symptom scores along with their composite symptom scores for each group shown in Table 2.

**Table 2:** Emotional, alcohol, and composite symptom scores of four subgroups.

Score^a^	Reference group	Group A	Group B	Group C	Total
Emotional symptom score (z)	0.00 (0.95)	5.07 (2.85)	0.12 (0.98)	5.47 (2.71)	1.57 (2.93)
HDRS (score)	2.61 (2.10)	13.74 (6.03)	2.73 (2.13)	14.49 (5.31)	6.01 (6.33)
HARS (score)	1.95 (1.86)	10.96 (5.59)	2.29 (1.93)	11.79 (5.83)	4.77 (5.47)
Alcohol-use symptom score (z)	0.00 (1.00)	−0.20 (1.08)	5.80 (2.84)	6.79 (3.26)	1.64 (3.32)
AUDIT (score)	2.33 (2.03)	1.92 (2.20)	14.13 (5.77)	16.13 (6.62)	5.67 (6.76)
Composite symptom score (z)	0.00 (0.70)	2.44 (1.43)	2.96 (1.56)	6.13 (2.18)	1.60 (2.40)

Data are presented as mean (standard deviation). Groups were defined as: Reference group (no symptoms), Group A (emotional symptoms only), Group B (alcohol-use symptoms only), and Group C (both symptoms).

AUDIT, Alcohol Use Disorder Identification Test; HARS, Hamilton Anxiety Rating Scale; HDRS, Hamilton Depression Rating Scale.

a The emotional symptom score is the mean of standardized z-scores from the HDRS and HARS scales. The alcohol-use symptom score is the standardized z-score of the AUDIT. The composite symptom score is the mean of the emotional and alcohol-use symptom scores. All z-scores are calculated using the means and standard deviations of the corresponding raw scores in the Reference group.

The frequencies of cardiovascular risk factors are reported in Supplementary Table 3.

### 3.2 Group differences in brain age gap

The validity of the current brain age prediction model was supported by an acceptable MAE of 2.787 years. Additionally, the model demonstrated a strong correlation between brain age and chronological age (n = 520, r = 0.896, p < 0.001, R^2^ = 0.803) (Supplementary Fig. 1).

**Fig. 1:**
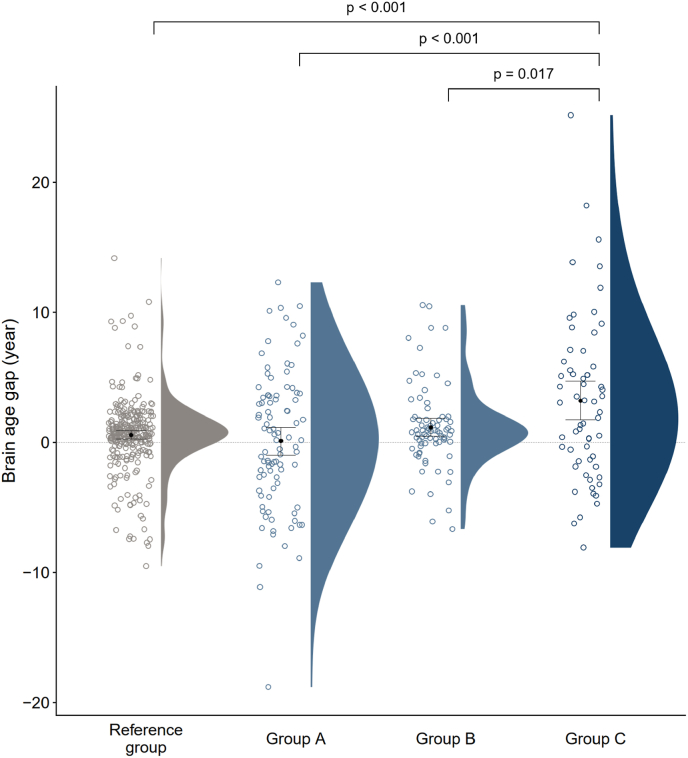
Brain age gap across the four subgroups. Groups were defined as: Reference group (no symptoms), Group A (emotional symptoms only), Group B (alcohol-use symptoms only), and Group C (both symptoms). Individual brain age gaps are shown as dots, with violin plots displaying distributions. Each group's mean is represented by a dot, with horizontal error bars showing 95 % confidence intervals. P-values were obtained from post-hoc pairwise comparisons using the Benjamini–Hochberg correction, following a two-way ANCOVA with chronological age as a covariate.

ANCOVA identified a significant main effect of having alcohol-use symptoms on the BAG (F (1,515) = 11.69, p < 0.001), while the effect of emotional symptom presence showed a trend toward significance (F (1,515) = 3.70, p = 0.055). Notably, a significant interaction was observed between emotional and alcohol-use symptoms on the BAG (F (1,515) = 5.72, p = 0.017). Post-hoc analyses showed that women with both emotional and alcohol-use symptoms had significantly greater BAG (mean ± SD = 3.20 ± 6.28 years) compared to those in the Reference group (0.56 ± 2.86 years), Group A (0.11 ± 5.36 years), and Group B (1.15 ± 3.19 years) (corrected p < 0.05) (Fig. 1).

### 3.3 Association between symptom severity and brain age gap

**Fig. 2:**
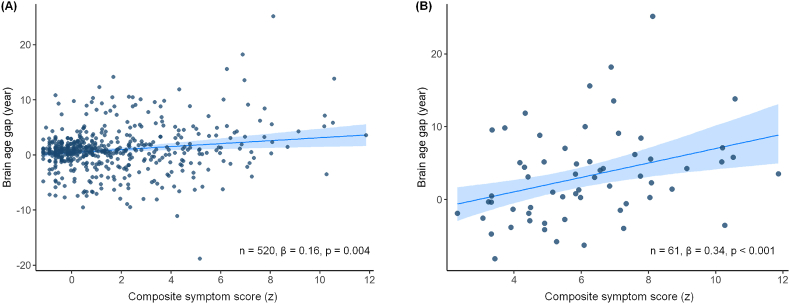
Associations between brain age gap and (A) composite symptom score across all participants or (B) within individuals with both emotional and alcohol-use symptoms. Chronological age was controlled for in the multiple linear regression analysis with bootstrapping. Regression coefficients are reported as standardized beta. Solid lines indicate regression lines, and shaded areas denote bootstrapped 95 % confidence intervals.

Across the full sample (n = 520), higher composite symptom scores were significantly associated with greater BAG after adjusting for chronological age (β = 0.16, p = 0.004) (Fig. 2A). Within the Group C (n = 61), which exhibited significantly elevated BAG compared to the other groups, further analysis revealed a significant positive association between composite symptom scores and BAG (β = 0.34, p < 0.001) (Fig. 2B).

### 3.4 Association between resilience levels and brain age gap

CD-RISC scores differed significantly across the groups (n = 516, F (3,512) = 35.77, p < 0.001). Post-hoc analyses revealed that the Group A (mean ± SD = 48.96 ± 16.29, n = 90) and Group C (46.82 ± 18.96) groups exhibited lower CD-RISC scores than the Reference group (64.54 ± 15.89, n = 286) and Group B (63.49 ± 15.21) (corrected p < 0.001).

**Fig. 3:**
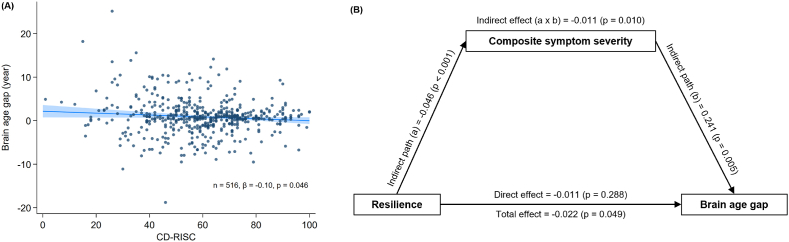
Association between resilience and brain age gap, and its mediation by symptom severity. (A) A scatterplot shows a significant negative association between the brain age gap and CD-RISC score across all participants, after controlling for chronological age. Regression coefficients are reported as standardized beta. The solid line represents the linear regression fit, and the shaded area indicates the 95 % bootstrapped confidence interval. (B) The mediation model demonstrates that the severity of composite emotional and alcohol-use symptoms significantly mediates the negative relationship between resilience and brain age gap. Coefficients and corresponding p values estimated via bootstrapping are shown for all paths. CD-RISC, Connor-Davidson Resilience Scale.

In the full sample (n = 516), lower resilience levels, as measured by CD-RISC, were significantly associated with greater BAG after adjusting for chronological age (β = −0.10, p = 0.046) (Fig. 3A). This association was not evident within individual groups (Reference group: β = −0.08, p = 0.150; Group A: β = 0.001, p = 0.988; Group B: β = 0.03, p = 0.756; Group C: β = −0.12, p = 0.362).

The mediation analysis examining the relationships between resilience, symptom severity, and BAG revealed that a negative association between CD-RISC and BAG was statistically mediated by composite symptom score (Fig. 3B, indirect effect = −0.011, p = 0.010, bootstrapped 95 % confidence interval [−0.019, −0.003]).

### 3.5 Effects of potential confounding factors

Sensitivity analyses re-examined the significance of the interaction between emotional and alcohol-use symptoms on BAG, the correlations of BAG with composite symptom score or resilience, and the statistical mediation of the resilience–BAG association by the composite symptom score, accounting for potential confounders including SES, cardiovascular risk factors, and types of SLE. Across sensitivity analyses, the results remained largely unchanged when each confounder was included as an additional covariate (Supplementary Table 4–7).

Furthermore, to assess the robustness of our findings, sensitivity analyses were performed by repeating the primary analyses after excluding 22 participants from the Reference group who had psychiatric diagnoses despite scoring below the established symptom threshold criteria on the HDRS, HARS, and AUDIT scales or one participant with a BAG value greater than 20 years. These analyses produced results that were largely consistent with the key findings (see Supplementary Results).

### 3.6 Brain age gap according to SLE exposure

We assessed whether BAG significantly differed according to specific types of SLE exposure. The analysis revealed that BAG did not significantly differ by types of SLE, although individuals exposed to traumatic events exhibited a marginal significant trend toward higher BAG compared to those who experienced other types of stressors (z = 1.87, p = 0.062).

We further evaluated differences in BAG as a function of the number of SLE types experienced. Among the 520 participants, 373 reported experiencing one SLE category, 147 reported two or more categories. As demonstrated in Supplementary Table 9, BAG did not significantly differ between these two groups (z = 0.29, p = 0.769).

## 4 Discussion

This study investigated the associations of emotional and alcohol-use symptoms, along with resilience, with brain aging in women experiencing recent SLE, utilizing an advanced brain age prediction model. Participants with both emotional and alcohol-use symptoms demonstrated higher BAG compared to other groups, with a significant interaction observed between the two symptoms. Composite symptom levels, encompassing both emotional and alcohol-use symptoms continuously, were positively associated with BAG in the full sample and within the subgroup with both symptoms. Conversely, resilience levels exhibited a negative association with BAG through statistical mediation by composite symptom severity. These findings suggest that emotional and alcohol-use symptoms may be jointly associated with accelerated brain aging among women in a more-than-additive manner, while resilience may be related to a potential buffering association. This study not only advances our understanding of the associations of stress-related symptoms and resilience with brain aging but also provides insights for developing management strategies for stress and related brain aging in women.

As shown in Table 1, our participants experienced various types of SLE. Personal and family-related stressors were the most prevalent, followed by traumatic events; however, these two types of stressors exhibited distinct patterns across the subgroups. The frequency of individuals reporting personal and family-related stress was highest in the Reference group, followed by the Group B, whereas traumatic stress was more common in the Group A and Group C. Although less prevalent overall, work and financial stressors as well as social and environmental stressors followed similar patterns to traumatic events. It is notable that individuals in the Group B reported higher levels of personal and family-related stress compared to those in the Group A and Group C. This finding may be explained by the lower prevalence of psychiatric diagnoses observed in the Group B. While alcohol use disorder were more prevalent in the Group B and Group C than in other groups, only a small proportion of participants in the Group B were diagnosed with any mental health disorders. In contrast, approximately half of the participants in the Group A and Group C were diagnosed with anxiety or depressive disorders.

The observed associations between advanced brain aging and emotional and alcohol-use symptoms align with prior research on stress-related brain aging. For example, Hatton et al. reported that alcohol consumption, low socioeconomic status, ethnicity, and cardiovascular risk factors were significantly associated with greater BAG in men (Hatton et al., 2018). Additionally, even after adjusting for these factors, a higher number of SLE correlated with increased predicted brain age relative to chronological age (Hatton et al., 2018). Longitudinal research further demonstrated that recent stress exposure was linked to accelerated brain aging, as well as anxiety and mood dysregulation, from the early to late 20s, regardless of sex (Mareckova et al., 2023). These findings support our results, suggesting that stress-related emotional and alcohol-use symptoms may be independently associated with brain aging, even in the absence of a diagnosed mental disorder.

Previous studies have shown that individuals with emotional or alcohol use disorders exhibit larger BAG compared to healthy controls (Blake et al., 2023; Guggenmos et al., 2017; Han et al., 2021b; Scheffler et al., 2024). A recent meta-analysis, for instance, reported an increased BAG of 0.90 years in patients with major depressive disorder (Blake et al., 2023). Similarly, individuals with anxiety or alcohol use disorders presented significantly larger BAG, approximately 3–4 years, relative to healthy individuals (Guggenmos et al., 2017; Han et al., 2021b; Scheffler et al., 2024). Although direct comparisons across studies are limited by methodological differences in sample characteristics, input features, and machine learning models, the increased BAG observed in the present study were smaller than those previously reported (Blake et al., 2023; Guggenmos et al., 2017; Han et al., 2021b; Scheffler et al., 2024). This discrepancy may be attributable to the broader, non-clinical sample used in this study, which was not restricted to patients with diagnosed psychiatric disorders. Nonetheless, the pronounced main effect of alcohol-use symptoms on BAG, contrasted with the marginal significance of emotional symptoms, is consistent with prior evidence suggesting a stronger influence of alcohol use on brain aging.

In this study, a positive association was observed between the combined levels of emotional and alcohol-use symptoms and BAG across all participants. The Group C exhibited significantly higher BAG than other groups, with a notable interaction effect between emotional and alcohol-use symptoms. These findings align with prior research suggesting that individuals with comorbid emotional and alcohol use disorders experience more pronounced brain deficits compared to those with only one condition (Miguel-Hidalgo et al., 2002; Sameti et al., 2011; Woodward et al., 2006). For instance, patients with PTSD and comorbid alcoholism displayed greater hippocampal volume loss compared to those with PTSD alone (Woodward et al., 2006). Similarly, long-term abstinent alcoholics with co-occurring emotional or externalizing disorders showed reduced volumes in the nucleus accumbens and hippocampus compared to those without such comorbidities (Sameti et al., 2011). Additionally, severe glial pathology in the prefrontal cortex has been observed in alcohol-dependent patients particularly when depressive symptoms were present (Miguel-Hidalgo et al., 2002). The observed synergistic and main effects of emotional and alcohol-use symptoms in this study suggest that these factors may be independently and interactively linked to accelerated brain aging. Clinically, these findings underscore the importance of monitoring brain aging and related functional declines in individuals with both symptoms.

While accelerated brain aging has been reported in several studies involving individuals with stress exposure or psychiatric disorders, the underlying neurobiological mechanisms remain to be fully elucidated. One potential mechanism involves stress-induced alterations in immune system gene expression, characterized by upregulation of proinflammatory genes and downregulation of genes involved in antibody production (Cole, 2014). This imbalance may lead to chronic inflammation, tissue damage, disrupted metabolic functions, and an increased risk of chronic and age-related diseases (Cole, 2014). Furthermore, impaired DNA repair mechanisms associated with chronic stress can exacerbate DNA damage, potentially resulting in neurodegeneration and accelerated brain aging (Ropert et al., 2024). The role of glucocorticoid signaling pathways in mediating faster epigenetic aging in response to cumulative lifetime stress also warrants further investigation (Zannas et al., 2015). Future research could explore potential associations between these neurobiological pathways and BAG estimates.

Across the full sample, higher levels of resilience were associated with smaller BAG. Results from the exploratory mediation analysis revealed that this association was mediated by composite symptom severity, indicating that lower resilience may contribute to elevated BAG indirectly through increased symptom burden. Although much attention has been given to risk factors such as stress and psychiatric disorders in brain aging research, associations between resilience and brain aging remains relatively underexplored. Consistent with our findings, a recent study demonstrated a negative association between resilience and BAG in older adults (Gu et al., 2025). Neuroimaging evidence further suggests that resilience may attenuate structural and functional deficits associated with emotional or alcohol use disorders (Elton et al., 2021; Jeong et al., 2019; Lee et al., 2023). For example, higher resilience has been linked to reduced cortical thinning and hypometabolism associated with PTSD (Jeong et al., 2019; Lee et al., 2023). Elton and colleagues identified phenotypes related to risk phenotypes and resilience in alcohol use disorder, showing that resilience phenotypes mitigate the impact of risk phenotypes on the disorder's development (Elton et al., 2021). However, the cross-sectional design of the study precludes definitive causal inferences regarding these pathways; thus, the findings should be interpreted with caution and require validation in future longitudinal research. Notably, previous studies have indicated that resilience can be enhanced through targeted interventions (Abate et al., 2024). Therefore, future interventional studies assessing whether resilience-promoting strategies can decelerate or potentially reverse brain aging could hold considerable clinical significance.

Our complementary analyses revealed that BAG did not significantly vary across SLE type and accumulation. However, our study did not collect detailed information regarding the frequency or subjective severity of individual SLE, which limits our ability to comprehensively evaluate the impact of stressor severity on brain aging. Future research incorporating detailed assessments of individual SLE characteristics, including frequency, duration, and perceived severity, is essential to elucidate the potential relationships between specific stressor dimensions and brain aging. Furthermore, considering prior evidence of a positive association between the number of negative fateful life events in midlife and larger BAG in older men (Hatton et al., 2018), additional studies are warranted to examine the effects of life stressor accumulation over longer periods.

Several limitations should be considered when interpreting these findings. First, the current study exclusively focused on women, which limits the generalizability of our results. While this focus was motivated by prior studies indicating that women may have heightened vulnerability to the negative impact of stress on mental health (Edwards et al., 2023; Li and Graham, 2017; McManus et al., 2022), the design of our study does not permit a direct examination of sex differences in the effects of stress on brain age. Although the topic of sex differences in stress-induced brain aging remains underexplored, one recent study found no significant association between stress-induced brain aging and sex (Mareckova et al., 2023). Therefore, future studies that include both sexes are warranted to investigate potential sex-specific effects of stress-related factors on brain aging. Second, the cross-sectional design of this study limits causal inferences regarding the relationship between stress-related symptoms and brain aging. A recent longitudinal study indicated that maternal depression during pregnancy was associated with larger BAG in offspring in their early and late 20s (Mareckova et al., 2023). Additionally, the study found that recent stress levels were associated with a faster pace of brain aging between these two timepoints, suggesting that pre-existing vulnerability and recent stress may differentially contribute to brain aging (Mareckova et al., 2023). To better understand these temporal dynamics, future longitudinal studies needed to elucidate the relationship between changes in stress and related symptoms, and the progression of brain aging. Third, while our findings on the association between composite symptom severity and BAG highlight the potential relevance of subclinical symptoms to brain aging, their generalizability to clinical populations should be interpreted with caution. This is primarily because our sample included only a small proportion of participants with a formal diagnosis of alcohol use disorder, warranting further validation in clinical cohorts. Fourth, brain aging is a complex biological process that may not be fully captured through structural imaging alone. Future research should prioritize the development, standardization, and application of more comprehensive prediction models that integrate multimodal neuroimaging data and other biological markers of aging to advance brain age research (Cole et al., 2019; Jirsaraie et al., 2023).

In conclusion, this study suggests that emotional and alcohol-use symptoms are associated with greater BAG in women experiencing SLE, independent of formal psychiatric diagnoses. Importantly, the co-occurrence of these symptoms was associated with an interaction consistent with BAG greater than the sum expected from their individual associations. Furthermore, stress-related symptom severity was positively associated with BAG, whereas resilience was negatively associated with BAG, potentially through the statistical mediation of symptom severity. These findings highlight potential distinct and interactive patterns involving emotional and alcohol-use symptoms, as well as resilience, in relation to BAG, and underscore the importance of considering stress-related brain aging in clinical assessment and management.

## CRediT authorship contribution statement

**Hyeonseok Jeong:** Writing – review & editing, Writing – original draft, Methodology, Formal analysis. **Yoonji Joo:** Writing – review & editing, Writing – original draft, Methodology, Investigation, Formal analysis, Data curation. **Youngeun Shim:** Writing – review & editing, Investigation, Data curation. **Yejin Kim:** Writing – review & editing, Investigation, Data curation. **Hyeonji Lee:** Writing – review & editing, Investigation, Data curation. **Yunjung Jin:** Writing – review & editing, Investigation, Data curation. **Seog Ju Kim:** Writing – review & editing, Investigation. **Sujung Yoon:** Writing – review & editing, Writing – original draft, Supervision, Resources, Project administration, Methodology, Funding acquisition, Formal analysis, Conceptualization. **In Kyoon Lyoo:** Writing – review & editing, Writing – original draft, Supervision, Resources, Project administration, Methodology, Funding acquisition, Formal analysis, Conceptualization.

## Funding

This research was supported by the 10.13039/501100003725National Research Foundation of Korea funded by the Korean government (RS-2024-00457381, RS-2024-00440371, and RS-2024-00345975).

## Declaration of competing interest

The authors declare the following financial interests/personal relationships which may be considered as potential competing interests:In Kyoon Lyoo reports financial support was provided by 10.13039/501100003725National Research Foundation of Korea. Sujung Yoon reports financial support was provided by 10.13039/501100003725National Research Foundation of Korea. If there are other authors, they declare that they have no known competing financial interests or personal relationships that could have appeared to influence the work reported in this paper.

## Data Availability

The data supporting the findings of this study are available from the corresponding author upon reasonable request. The data are not publicly available due to privacy and ethical restrictions.
