# Supplementary material for: Potential vulnerability and resilience to accelerated brain aging in women exposed to stressful life events: insights from the brain age prediction model
Source: Neurobiol Stress. 2025 Sep 23;39:100763. doi: 10.1016/j.ynstr.2025.100763 (PMC12508567; doi:10.1016/j.ynstr.2025.100763)
Supplement: Multimedia component 1 [file mmc1.docx]

**Supplementary Material for**

**Potential vulnerability and resilience to accelerated brain aging in women exposed to stressful life events: insights from the brain age prediction model**

Hyeonseok Jeong, Yoonji Joo, Youngeun Shim, Yejin Kim,

Hyeonji Lee, Yunjung Jin, Seog Ju Kim, Sujung Yoon, In Kyoon Lyoo

Correspondence: sujungjyoon@ewha.ac.kr (S.Y.) and inkylyoo@ewha.ac.kr (I.K.L.)

**Supplementary Results**

**Sensitivity analysis excluding participants with psychiatric diagnoses in the Reference group**

To evaluate whether our primary findings were influenced by the presence of participants with formal psychiatric diagnoses in the Reference group (n = 22), we conducted comprehensive sensitivity analyses by repeating all statistical procedures after excluding these individuals. The statistical significance and directionality of the key results remained largely consistent. Specifically, in the two-way ANCOVA testing the main and interaction effects of emotional and alcohol-use symptoms with age as a covariate, the main effects of both emotional symptoms (F(1, 493) = 4.40, p = 0.036) and alcohol-use symptoms (F(1, 493) = 13.02, p < 0.001) were significant. The interaction between these symptoms was also significant (F(1, 493) = 4.95, p = 0.027).

In addition, across all participants, composite symptom scores were positively correlated with BAG after adjusting for age (β = 0.17, p = 0.002), whereas CD-RISC scores exhibited a trend toward a negative association (β = -0.09, p = 0.054). In the mediation analysis, the indirect effect of composite symptom severity linking resilience to BAG was significant (b = -0.012, p = 0.008).

**Sensitivity analysis excluding potential outliers with extreme brain age gap values**

To examine whether the main findings were driven by extreme BAG values (greater than 20 years or less than -20 years), additional sensitivity analyses were performed by repeating all statistical analyses after excluding one participant with BAG > 20 years. The statistical significance and directionality of our main results remained largely consistent.

The main effect of alcohol-use symptoms remained significant (F(1,514) = 9.93, p = 0.002), whereas the main effect of emotional symptoms did not (F(1,514) = 2.39, p = 0.123), mirroring the original two-way ANCOVA model testing the main and interaction effects of emotional and alcohol-use symptom presence with age as a covariate. The interaction between the two symptom domains also remained significant (F(1, 514) = 4.27, p = 0.039).

In addition, composite symptom scores were positively related to BAG across the full sample after adjusting for age (β = 0.14, p = 0.005), whereas CD-RISC scores showed a trend toward a negative association (β = -0.08, p = 0.088). The mediation analysis revealed the significant indirect effect of composite symptom severity between resilience and BAG (b = -0.008, p = 0.018).

**Supplementary Table 1.** Demographic characteristics of the dataset used for retraining the brain age prediction model

| Dataset | n (%) | Age | Male |
| --- | --- | --- | --- |
|  |  | mean ± SD (range) | n (%) |
| Training set | 3,269 (80.0%) | 39.7 ± 13.4 (18.6 to 79.8) | 1,520 (46.5%) |
| Test set | 818 (20.0%) | 39.8 ± 13.4 (19.4 to 79.5) | 382 (46.7%) |
| Total | 4,087 (100.0%) | 39.7 ± 13.4 (18.6 to 79.8) | 1,902 (46.5%) |

The dataset comprised 4,087 T1-weighted imaging datasets, randomly divided into training and test sets in an 8:2 ratio.

| Diagnosis^a^ | Reference  group  (n =287) | Group A  (n = 93) | Group B  (n = 79) | Group C  (n = 61) | Total  (n = 520) | p |
| --- | --- | --- | --- | --- | --- | --- |
| Major depressive disorder | 0 (0.0%) | 30 (32.3%) | 0 (0.0%) | 26 (42.6%) | 56 (10.8%) | < 0.001 |
| Dysthymic disorder | 9 (3.1%) | 5 (5.4%) | 3 (3.8%) | 3 (4.9%) | 20 (3.9%) | 0.657 |
| Panic disorder | 0 (0.0%) | 6 (6.5%) | 1 (1.3%) | 4 (6.6%) | 11 (2.1%) | < 0.001 |
| Obsessive compulsive disorder | 0 (0.0%) | 0 (0.0%) | 0 (0.0%) | 1 (1.6%) | 1 (0.2%) | 0.117 |
| Posttraumatic stress disorder^b^ | 11 (3.9%) | 38 (40.9%) | 4 (5.1%) | 30 (49.2%) | 83 (16.0%) | < 0.001 |
| Generalized anxiety disorder | 0 (0.0%) | 2 (2.2%) | 0 (0.0%) | 1 (1.6%) | 3 (0.6%) | 0.043 |
| Specific phobia | 0 (0.0%) | 2 (2.2%) | 0 (0.0%) | 0 (0.0%) | 2 (0.4%) | 0.068 |
| Alcohol use disorder | 0 (0.0%) | 0 (0.0%) | 4 (5.1%) | 4 (6.6%) | 8 (1.5%) | < 0.001 |
| Bipolar I disorder | 1 (0.4%) | 0 (0.0%) | 0 (0.0%) | 1 (1.6%) | 2 (0.4%) | 0.330 |
| Bulimia nervosa | 1 (0.4%) | 1 (1.1%) | 0 (0.0%) | 0 (0.0%) | 2 (0.4%) | 0.696 |
| Binge eating disorder | 0 (0.0%) | 1 (1.1%) | 0 (0.0%) | 0 (0.0%) | 1 (0.2%) | 0.448 |

**Supplementary Table 2.** Prevalence of psychiatric diagnoses

Data are presented as n (%). Groups were defined as: Reference (no symptoms), Group A (emotional symptoms only), Group B (alcohol-use symptoms only), and Group C (both symptoms).

^a^ Only diagnoses observed in at least one participant are presented. Group differences in the frequency of each diagnosis were evaluated using Fisher's exact tests.

^b^ The diagnostic status for one participant in the no-risk group was missing.

**Supplementary Table 3.** Cardiovascular risk factors of study participants

| Risk factors | Reference  group  (n =287) | Group A  (n = 93) | Group B  (n = 79) | Group C  (n = 61) | Total  (n = 520) | p |
| --- | --- | --- | --- | --- | --- | --- |
| Hypertension | 3 (1.1%) | 4 (4.3%) | 1 (1.3%) | 5 (8.2%) | 13 (2.5%) | 0.008^b^ |
| Hyperlipidemia | 5 (1.7%) | 4 (4.3%) | 3 (3.8%) | 4 (6.6%) | 16 (3.1%) | 0.124^b^ |
| Diabetes | 2 (0.7%) | 3 (3.2%) | 1 (1.3%) | 2 (3.3%) | 8 (1.5%) | 0.102^b^ |
| Obesity^a^ | 60 (21.0%) | 23 (24.7%) | 18 (22.8%) | 11 (18.0%) | 112 (21.6%) | 0.767^b^ |
| Smoking | 39 (13.6%) | 21 (22.6%) | 29 (36.7%) | 23 (37.7%) | 112 (21.5%) | < 0.001^b^ |
| Total number of risk factors^a^ | 0.4 (0.6) | 0.6 (0.8) | 0.7 (0.8) | 0.7 (1.0) | 0.5 (0.7) | < 0.001^c^ |

Data are presented as number (%) or mean (standard deviation). Groups were defined as: Reference (no symptoms), Group A (emotional symptoms only), Group B (alcohol-use symptoms only), and Group C (both symptoms).

^a^ One participant in the Reference group was not assessed.

^b^ Fisher's exact test.

^c^ Kruskal-Wallis test.

| Additional covariate in the main model | Main effect of alcohol-use symptoms | Main effect of emotional symptoms | Interaction effect between alcohol-use and emotional symptoms |
| --- | --- | --- | --- |
| None (main model) | F(1,515) = 11.69,  p < 0.001 | F(1,515) = 3.70,  p = 0.055 | F(1,515) = 5.72,  p = 0.017 |
| Socioeconomic status | F(1,513) = 11.03,  p = 0.001 | F(1,513) = 2.35,  p = 0.126 | F(1,513) = 5.88,  p = 0.016 |
| Cardiovascular risk factors |  |  |  |
| Hypertension | F(1,514) = 13.32,  p < 0.001 | F(1,514) = 5.07,  p = 0.025 | F(1,514) = 6.64,  p = 0.010 |
| Hyperlipidemia | F(1,514) = 11.22,  p < 0.001 | F(1,514) = 3.51,  p = 0.062 | F(1,514) = 5.64,  p = 0.018 |
| Diabetes | F(1,514) = 11.44,  p < 0.001 | F(1,514) = 3.36,  p = 0.068 | F(1,514) = 5.70,  p = 0.017 |
| Obesity^a^ | F(1,513) = 11.83,  p < 0.001 | F(1,513) = 3.66,  p = 0.056 | F(1,513) = 6.11,  p = 0.014 |
| Smoking | F(1,514) = 11.32,  p < 0.001 | F(1,514) = 3.69,  p = 0.055 | F(1,514) = 5.69,  p = 0.017 |
| Total number of risk factors^a^ | F(1,513) = 10.85,  p = 0.001 | F(1,513) = 3.40,  p = 0.066 | F(1,513) = 5.96,  p = 0.015 |
| Types of stressful life events |  |  |  |
| Personal and family-related stressors | F(1,514) = 11.77,  p = 0.001 | F(1,514) = 3.77,  p = 0.053 | F(1,514) = 5.65,  p = 0.018 |
| Work and financial stressors | F(1,514) = 11.64,  p = 0.001 | F(1,514) = 4.06,  p = 0.045 | F(1,514) = 5.52,  p = 0.019 |
| Social and environmental stressors | F(1,514) = 11.93,  p = 0.001 | F(1,514) = 4.30,  p = 0.039 | F(1,514) = 5.87,  p = 0.016 |
| Traumatic events | F(1,514) = 10.98,  p = 0.001 | F(1,514) = 1.71,  p = 0.191 | F(1,514) = 5.68,  p = 0.018 |

**Supplementary Table 4.** Sensitivity analyses for the ANCOVA model

Sensitivity analyses of the ANCOVA model evaluating the main and interactive associations of emotional and alcohol-use symptom presence with brain age gap were conducted by adding socioeconomic status, cardiovascular risk factors, or types of stressful life events individually as additional covariates in separate models.

^a^ One participant in the Reference group was not assessed.

| Additional covariate in the main model | Full sample | Group C |
| --- | --- | --- |
| None (main model) | β = 0.16, p = 0.004 | β = 0.34, p < 0.001 |
| Socioeconomic status | β = 0.15, p = 0.003 | β = 0.32, p = 0.002 |
| Cardiovascular risk factors |  |  |
| Hypertension | β = 0.17, p = 0.002 | β = 0.33, p = 0.002 |
| Hyperlipidemia | β = 0.16, p = 0.004 | β = 0.36, p < 0.001 |
| Diabetes | β = 0.16, p = 0.004 | β = 0.35, p < 0.001 |
| Obesity^a^ | β = 0.16, p = 0.004 | β = 0.34, p = 0.002 |
| Smoking | β = 0.16, p = 0.002 | β = 0.34, p < 0.001 |
| Total number of risk factors^a^ | β = 0.15, p = 0.004 | β = 0.34, p = 0.002 |
| Types of stressful life events |  |  |
| Personal and family-related stressors | β = 0.17, p = 0.001 | β = 0.37, p < 0.001 |
| Work and financial stressors | β = 0.16, p < 0.001 | β = 0.36, p < 0.001 |
| Social and environmental stressors | β = 0.17, p = 0.001 | β = 0.36, p = 0.001 |
| Traumatic events | β = 0.15, p = 0.011 | β = 0.31, p = 0.004 |

**Supplementary Table 5.** Sensitivity analyses of the association between brain age gap and the composite symptom score

Sensitivity analyses of the main regression model for the association between brain age gap and the CD-RISC score in the full sample. The analyses were repeated by separately adding socioeconomic status, cardiovascular risk factors, or types of stressful life events as an additional covariate to the model.

^a^ One participant in the Reference group was not assessed.

**Supplementary Table 6.** Sensitivity analyses of the association between brain age gap and resilience

Sensitivity analyses of the main regression model for the association between brain age gap and the CD-RISC score in the full sample. The analyses were repeated by separately adding socioeconomic status, cardiovascular risk factors, or types of stressful life event as an additional covariate to the model.

a One participant in the Reference group was not assessed.

CD-RISC, Connor-Davidson Resilience Scale.

| Additional covariate in the main model | Full sample |
| --- | --- |
| None (main model) | β = -0.10, p = 0.046 |
| Socioeconomic status | β = -0.08, p = 0.066 |
| Cardiovascular risk factors |  |
| Hypertension | β = -0.09, p = 0.047 |
| Hyperlipidemia | β = -0.09, p = 0.050 |
| Diabetes | β = -0.09, p = 0.049 |
| Obesity^a^ | β = -0.10, p = 0.047 |
| Smoking | β = -0.09, p = 0.046 |
| Total number of risk factors^a^ | β = -0.10, p = 0.046 |
| Types of stressful life events |  |
| Personal and family-related stressors | β = -0.10, p = 0.045 |
| Work and financial stressors | β = -0.10, p = 0.040 |
| Social and environmental stressors | β = -0.10, p = 0.043 |
| Traumatic events | β = -0.08, p = 0.072 |

**Supplementary Table 7.** Sensitivity analyses of the mediation model linking resilience, composite symptom score, and brain age gap

| Additional covariate in the main model | Indirect effect |
| --- | --- |
| None (main model) | b = -0.011, p = 0.010 |
| Socioeconomic status | b = -0.009, p = 0.016 |
| Cardiovascular risk factors |  |
| Hypertension | b = -0.012, p = 0.005 |
| Hyperlipidemia | b = -0.011, p = 0.013 |
| Diabetes | b = -0.011, p = 0.012 |
| Obesity^a^ | b = -0.011, p = 0.010 |
| Smoking | b = -0.011, p = 0.008 |
| Total number of risk factors^a^ | b = -0.010, p = 0.011 |
| Types of stressful life events |  |
| Personal and family-related stressors | b = -0.011, p = 0.010 |
| Work and financial stressors | b = -0.011, p = 0.008 |
| Social and environmental stressors | b = -0.012, p = 0.007 |
| Traumatic events | b = -0.009, p = 0.024 |

Sensitivity analyses of the mediation model examining the association between brain age gap and the CD-RISC score with the composite symptom score as a mediator in the full sample. The analyses were repeated by separately adding socioeconomic status, cardiovascular risk factors, or types of stressful life events as an additional covariate to the model.

^a^ One participant in the Reference group was not assessed.

CD-RISC, Connor-Davidson Resilience Scale.

**Supplementary Table 8.** Brain age gap by type of stressful life event

| Measure | Type of stressful life event | | Test |
| --- | --- | --- | --- |
|  | Personal and family-related stressors  (n = 381) | Other types  (n = 139) |  |
| Brain age gap (year) | 0.70 (3.56) | 1.36 (5.17) | z = -0.30, p = 0.767 |
|  |  |  |  |
|  | Work and financial stressors  (n = 68) | Other types  (n = 452) |  |
| Brain age gap (year) | 0.99 (5.46) | 0.86 (3.81) | z = -0.52, p = 0.600 |
|  |  |  |  |
|  | Social and environmental stressors  (n = 29) | Other types  (n = 491) |  |
| Brain age gap (year) | 1.56 (5.56) | 0.84 (3.96) | z = -0.17, p = 0.878 |
|  |  |  |  |
|  | Traumatic events  (n = 202) | Other types  (n = 318) |  |
| Brain age gap (year) | 1.38 (5.16) | 0.56 (3.14) | z = 1.87, p = 0.062 |

Data are presented as mean (standard deviation). Group comparisons were conducted separately for each category of stressful life events based on endorsement (yes/no). Because some participants met criteria for multiple categories, categories were not treated as mutually exclusive.

**Supplementary Table 9.** Brain age gap by number of SLE types

| Measure | Single SLE type  (n = 373) | Multiple SLE types  (n = 147) | Test |
| --- | --- | --- | --- |
| Brain age gap (year) | 0.83 (3.55) | 0.99 (5.14) | z = 0.29, p = 0.769 |

Data are presented as mean (standard deviation). Group comparisons were performed between participants with a single SLE type and those with multiple SLE types.

SLE, stressful life event.


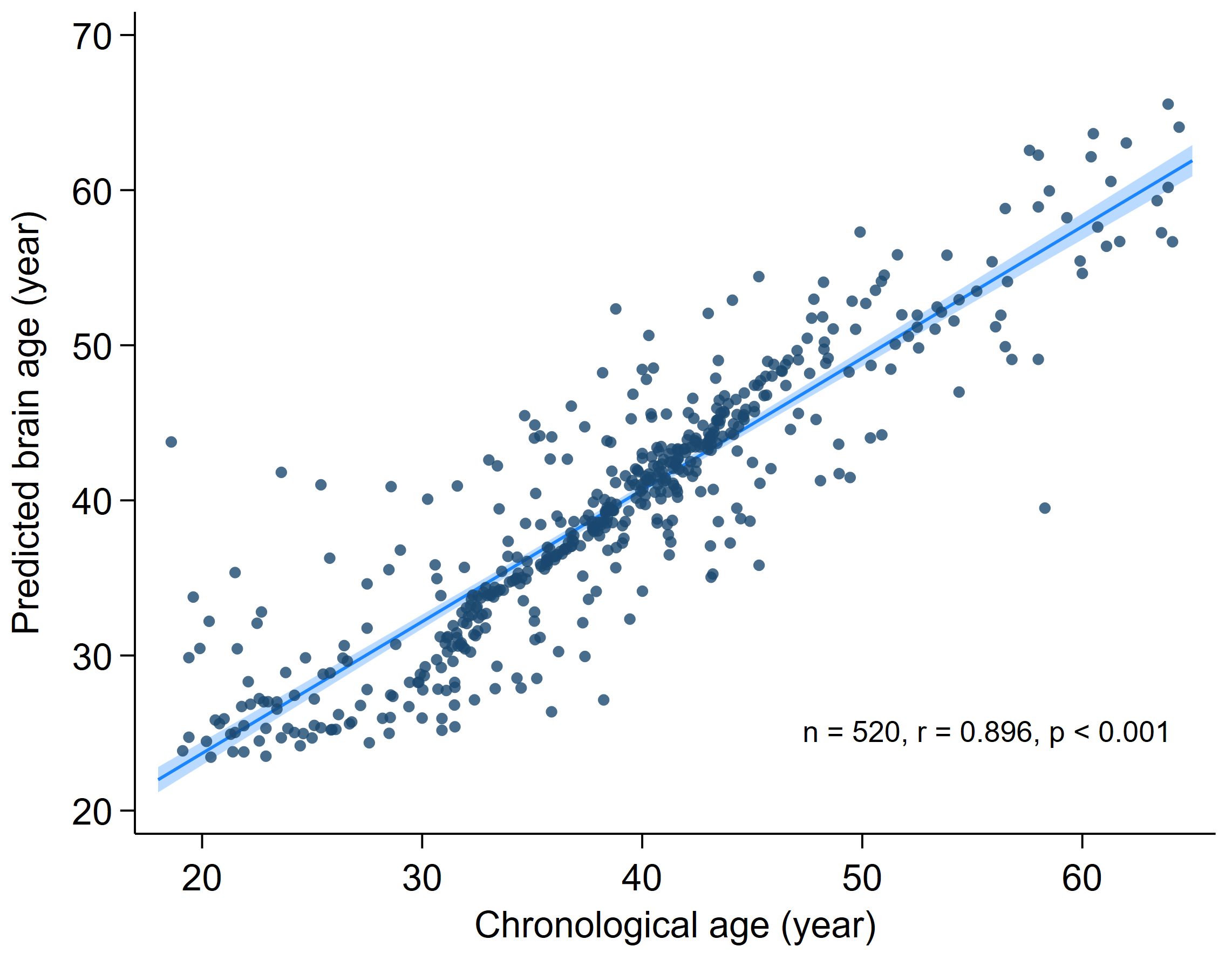


**Supplementary Fig. 1.** Association between predicted brain age and chronological age in study participants. The solid line and shaded area depict the regression line and 95% confidence interval, respectively.
